# Decreasing the tactile interaction between skin, sweat and clothing significantly reduces the perception of wetness independently of the level of physical skin wetness during moderate exercise

**DOI:** 10.1186/2046-7648-4-S1-A76

**Published:** 2015-09-14

**Authors:** Davide Filingeri, Damien Fournet, Simon Hodder, George Havenith

**Affiliations:** 1 Environmental Ergonomics Research Centre, Loughborough Design School, Loughborough University, Loughborough, UK; 2 Thermal Sciences Laboratory, Oxylane Research, Villeneuve d'Ascq, France

## Introduction

Although the ability to sense skin wetness and humidity is critical for behavioural and autonomic adaptations, humans are not provided with specific skin receptors for sensing wetness [1]. We have recently demonstrated that humans perceive the wetness experienced when the skin is in contact with a wet surface through a multisensory integration of thermal and tactile inputs generated by the interaction between skin and moisture [2]. To further the understanding on the neurophysiology of human skin wetness perception, here we tested the hypothesis that the perception of sweat-induced skin wetness can be significantly manipulated, independently from the level of physical skin wetness.

## Methods

Ten males (mean age (SD) 22 (2) years, height 180.3 (6) cm, body mass 79.6 (10) kg) repeated an incremental walking protocol (5 km.h^-1^; gradient range: +2 to +16 %) during two trials designed to produce the same level of physical skin wetness, but to induce lower (i.e. TIGHT-FIT) and higher (i.e. LOOSE-FIT) perception of wetness. During the TIGHT-FIT trial, a tight fitting clothing ensemble was worn to limit the mechanical interaction and stickiness between skin, sweat and clothing. During the LOOSE-FIT trial, a loose fitting ensemble was used to augment this interaction. To limit the amount of moisture evaporation from the skin (and thus skin cooling), a vapour impermeable, loose fitting clothing ensemble was worn as a second layer on top of both the loose or the tight fitting garments. Heart rate, rectal temperature, mean skin temperature, whole body skin wetness (*w*_body_) and galvanic skin conductance (GSC), as well as thermal, wetness and comfort sensation were recorded.

## Results

Both sweat production (indicated by GSC) and physical skin wetness (indicated by *w*_body_) increased significantly during the protocol (GSC range: 3.1 (0.3) to 18.8 (1.3) µS, p < 0.01; *w*_body _range: 0.26 (0.01) to 0.95 (0.2)nd (non-dimension unit), p < 0.01) with no differences between TIGHT-FIT and LOOSE-FIT (p > 0.05). However, the reduced skin friction generated by the TIGHT-FIT ensemble lowered significantly the level of perceived skin wetness, both at a whole-body and at a regional level (p < 0.01). Regression analyses performed between indicators of physical wetness (i.e. *w*_body _and mean GSC) and perceived skin wetness indicated that when *w*_body _ranged from ~0.4 to ~0.8 nd and when mean GSC ranged from ~4.5 to ~9.5 µS, skin wetness perception was significantly reduced when wearing tight as opposed to loose fitting garments.

## Conclusion

We conclude that under conditions of sweat-induced whole-body wetness and of absence of skin cooling, the perception of skin wetness is primarily driven by the degree of tactile interaction between skin, sweat and clothing, and that by manipulating this interaction (e.g. changing the clothing fit) skin wetness perception can be significantly altered, independently of the level of physical wetness.

## References

[B1] Clark R Edholm O Man and his thermal environment 1985 London: E. Arnold

[B2] Filingeri D Why wet feels wet? A neurophysiological model of human cutaneous wetness sensitivity J Neurophysiol 2014 doi: 10.1152/jn.00120.2014 10.1152/jn.00120.2014 24944222

